# Targeting Persistent Changes in Neuroimmune and Epigenetic Signaling in Adolescent Drinking to Treat Alcohol Use Disorder in Adulthood

**DOI:** 10.1124/pharmrev.122.000710

**Published:** 2023-03

**Authors:** Fulton T. Crews, Leon G. Coleman, Victoria A. Macht, Ryan P. Vetreno

**Affiliations:** Bowles Center for Alcohol Studies and Departments of Pharmacology and Psychiatry, School of Medicine, University of North Carolina School of Medicine, Chapel Hill, North Carolina

## Abstract

Studies universally find early age of drinking onset is linked to lifelong risks of alcohol problems and alcohol use disorder (AUD). Assessment of the lasting effect of drinking during adolescent development in humans is confounded by the diversity of environmental and genetic factors that affect adolescent development, including emerging personality disorders and progressive increases in drinking trajectories into adulthood. Preclinical studies using an adolescent intermittent ethanol (AIE) exposure rat model of underage binge drinking avoid the human confounds and support lifelong changes that increase risks. AIE increases adult alcohol drinking, risky decision-making, reward-seeking, and anxiety as well as reductions in executive function that all increase risks for the development of an AUD. AIE causes persistent increases in brain neuroimmune signaling high-mobility group box 1 (HMGB1), Toll-like receptor, receptor for advanced glycation end products, and innate immune genes that are also found to be increased in human AUD brain. HMGB1 is released from cells by ethanol, both free and within extracellular vesicles, that act on neurons and glia, shifting transcription and cellular phenotype. AIE-induced decreases in adult hippocampal neurogenesis and loss of basal forebrain cholinergic neurons are reviewed as examples of persistent AIE-induced pathology. Both are prevented and reversed by anti-inflammatory and epigenetic drugs. Findings suggest AIE-increased HMGB1 signaling induces the RE-1 silencing transcript blunting cholinergic gene expression, shifting neuronal phenotype. Inhibition of HMGB1 neuroimmune signaling, histone methylation enzymes, and galantamine, the cholinesterase inhibitor, both prevent and reverse AIE pathology. These findings provide new targets that may reverse AUD neuropathology as well as other brain diseases linked to neuroimmune signaling.

**Significance Statement:**

Adolescent underage binge drinking studies find that earlier adolescent drinking is associated with lifelong alcohol problems including high levels of lifetime alcohol use disorder (AUD). Preclinical studies find the underage binge drinking adolescent intermittent ethanol (AIE) model causes lasting changes in adults that increase risks of developing adult alcohol problems. Loss of hippocampal neurogenesis and loss of basal forebrain cholinergic neurons provide examples of how AIE-induced epigenetic and neuroimmune signaling provide novel therapeutic targets for adult AUD.

## I. Introduction: Adolescent and Underage Drinking are Common But Differ From AUD

Adolescent alcohol drinking contributes to long-lasting changes in adults that increase risks for adult problem drinking and alcohol use disorder (AUD). AUD has behavioral, cognitive, and physiologic symptoms related to repeated heavy alcohol use, a strong desire to consume alcohol, and difficulties in controlling its use despite problems that can include withdrawal symptoms and blackouts (Schuckit, 2009; Tavolacci et al., 2019). Adolescent drinking differs from the daily heavy drinking in adults with AUD; adolescents generally drink on social occasions and not every day. Adolescents tend to drink more alcohol per occasion than adults, with high levels of binge drinking, which in the United States is related to the legal driving limit blood level of 80 mg%, which represents about five or more drinks for males and four or more for females within 2 hours. One study found that 10% of high school seniors endorse extreme binge drinking of 10 or more drinks in a row, with 5.6% reporting 15 or more drinks in a row (Patrick et al., 2013). Extreme binge drinking likely has blood levels over 200 mg% that are linked to acute loss of memory (i.e., blackouts) from high blood alcohol levels (Hartzler and Fromme, 2003; Wetherill and Fromme, 2009; Rose and Grant, 2010). Similarly, evidence suggests that the developing adolescent cortex is more sensitive to alcohol-induced damage than the adult brain (Crews et al., 2000; Crews et al., 2007). There are also long-term consequences of adolescent drinking, including increased lifelong risks of alcohol-related problems and AUD. However, the discrete effects of adolescent binge drinking that persist for a lifetime are poorly understood.

## II. Age of Drinking Onset and Risks for AUD

Studies universally agree that an early adolescent age of drinking onset or initiation leads to increased adolescent and lifelong alcohol drinking, alcohol-related problems, and risks for development of an AUD (Grant and Dawson, 1997; Grant et al., 2001a; McGue et al., 2001; Hingson et al., 2006; Pitkanen et al., 2008; Lodha et al., 2022). Although early adolescent drinking is rare, sipping alcohol in sixth grade with parents is found to increase the chances of getting drunk and drinking heavily by ninth grade compared with nonsippers (Jackson et al., 2015). A longitudinal study of 800 individuals from 11 to 18 years of age found that a younger age of alcohol initiation is a major factor related to a higher level of alcohol misuse at ages 17 and 18 (Hawkins et al., 1997). Several large-scale human studies, such as the Swedish study of 8000 twins following individuals who started drinking at age 10 (Prescott and Kendler, 1999) or a United States study of families following individuals from age 14 (Grant and Dawson, 1997; Grant et al., 2001b), find the youngest age groups had a 40% to 50% lifetime prevalence of AUD, which decline to a 10% lifetime prevalence of AUD if drinking initiation started at or after age 20. A younger adolescent age of drinking onset also increases the risk for lifetime other drug dependence, violence, and fights and injuries associated with alcohol use (Grant and Dawson, 1997; Sher and Gotham, 1999; DeWit et al., 2000; Grant et al., 2006; Hingson et al., 2009). These studies suggest early adolescent drinking may be particularly important; however, a French study comparing 25- to 45-year-old adults with AUD and similar-aged controls who did not initiate drinking until about 15 years of age, but binge drank between 18 and 25 years of age, finds that late adolescent binge drinking can also lead to later adult alcohol dependence (Tavolacci et al., 2019). Thus, studies all agree that adolescent binge drinking is linked to adult alcohol problems and risks for AUD.

The effect of adolescent drinking in many adult studies is confounded by variations in human genes, diet, adolescent onset of mental disease, and family environment as well as the common trajectory of increased drinking with age from adolescence to adult heavy drinking and development of AUD. Since initiation of alcohol drinking in early adolescence typically continues into adulthood, adult drinking confounds a clear understanding of the effect of an early age of drinking onset. To understand the effect of an early age of drinking onset, it is important to distinguish the effects of adolescent alcohol exposure on maturation of adult neurobiology and behavior from the effect of AUD and heavy drinking in adults. If adolescent drinking creates unique lifelong risks as studies suggest, this supports focused prevention efforts in early adolescence. Interestingly, studies in rats find early adolescence is a more sensitive period (Alaux-Cantin et al., 2013; Spear, 2015), similar to the higher risks with earlier ages of drinking onset in human. Preclinical studies allow direct testing of hypotheses that adolescent alcohol exposure leads to lifelong lasting changes independent of adult drinking.

## III. Adolescent Intermittent Ethanol Exposure Procedure Models Human Underage Weekend Binge Drinking Exposure and Persistent Changes in Adulthood

Adolescent alcohol drinking is characterized as binge and extreme drinking, typically occurring during social events and vacations, that differs from the daily heavy drinking in adults with alcohol dependence. In 2007, Maria Pascual and Consuelo Guerri recognized the need to develop a preclinical underage binge drinking model in Wistar rats and designed the adolescent intermittent ethanol (AIE) procedure involving eight ethanol treatments (3 gm/kg, 25% i.p., BEC > 200 mg%) across puberty [postnatal days (P) 25–40], with determinations in late adolescence just after the last ethanol treatment, as well as weeks later in adulthood, to test hypotheses on the lasting effect of adolescent binge drinking on adult brain. They discovered increased levels of proinflammatory iNOS and COX2 just after AIE treatment as well as following 3 weeks of abstinent maturation to adulthood (Pascual et al., 2007). AIE also altered balance and exploratory behavior and indomethacin, the anti-inflammatory drug, prevented the AIE-induced increases in iNOS and COX2 as well as behaviors. Interest in adolescent research has increased, with AIE adopted by many groups. Preclinical studies on rodent alcohol drinking have found that rearing conditions, group versus single housing (Lodha et al., 2022), and chow diet (Marshall et al., 2015) as well as the amount and form of adolescent ethanol exposure affect responses and confound results(Crews et al., 2016). For example, studies find adolescent rats reared as a group together compared with socially isolated rearing show less adult anxiety-like behavior and lower adult ethanol self-administration. Adult rats from the vendor show high anxiety and ethanol drinking similar to the social isolation group (Chappell et al., 2013), illustrating the importance of controlling developmental confounding factors. AIE has been refined to improve replication by the Neurobiology of Adolescent Drinking in Adulthood (NADIA) consortium to standardize early life environment, rearing, and ages as well as binge BEC > 100 mg% exposure that includes ethanol vapor exposure, treatment of males and females across puberty, and adult assessments (Crews et al., 2016, 2019). Assessments are conducted in young adulthood to determine the lasting effect of adolescent alcohol exposure in adults without the confounds of continuing adult exposure. Also, adult characteristics are generally stable, allowing longitudinal studies and/or extended behavioral training without the confound of adolescent age-related changes or additional adult ethanol exposure. The AIE design focuses on the unique persistent effect of underage drinking on adult neurobiology that allows determination of how ethanol can change adult neurobiology independent of adult alcohol drinking.

### A. AIE and Adult Risk Factors for AUD

Multiple studies, including those from the NADIA consortium, report that AIE promotes adult alcohol drinking (Rodd-Henricks et al., 2002; Pascual et al., 2009; Alaux-Cantin et al., 2013; Broadwater et al., 2013; Gass et al., 2014; Vargas et al., 2014; Pandey et al., 2015; Toalston et al., 2015; Lee et al., 2017; Wille-Bille et al., 2017; Lodha et al., 2022). Adult drinking is increased after adolescent ethanol exposure in both adult sexes, with females drinking more than males (Amodeo et al., 2018). AIE drinking or AIE ethanol vapor exposure also increase adult operant responding for ethanol self-administration and reduce extinction (Gass et al., 2014; Amodeo et al., 2017), suggesting that adolescent ethanol exposure causes cognitive-behavioral shifts frequently associated with increased risk factors for developing AUD. AIE exposure also alters adult anxiety, behavioral flexibility, and responses to acute alcohol in adulthood, suggesting that the effects of adolescent drinking are widespread and affect multiple cognitive-behavioral domains. Learning studies find AIE causes little to no change in learning ability (Semenova, 2012; Risher et al., 2013; Gass et al., 2014; Mejia-Toiber et al., 2014; Boutros et al., 2016), although AIE impairs complex operant tasks that involve a rule change or set-shifting (Gass et al., 2014) and memory tasks such as spatial–temporal object recognition (Swartzwelder et al., 2015). Other studies using spatial learning tasks (i.e., the Morris water maze or the Barnes maze) find initial learning is intact and not altered, but changing the goal location in a reversal learning task reveals AIE-induced reversal deficiencies interpreted as behavioral inflexibility (Coleman et al., 2011; Vetreno and Crews, 2012; Acheson et al., 2013; Coleman et al., 2014; Vetreno et al., 2020) and loss of executive function (Crews et al., 2016). AIE increases risky choices (Schindler et al., 2014; Boutros et al., 2016; Schindler et al., 2016) and enhances reward-seeking in adulthood (McClory and Spear, 2014; Spoelder et al., 2015; Kruse et al., 2017; Madayag et al., 2017). Another effect of AIE is heightened social anxiety in adulthood (Varlinskaya and Spear, 2015), particularly in males (Varlinskaya et al., 2014, 2017; Dannenhoffer et al., 2018), following early AIE exposure as compared with late adolescent AIE exposure (Varlinskaya et al., 2014). Other studies reveal AIE increases anxiety-like behavior in adulthood using the elevated-plus maze (Pandey et al., 2015; Sakharkar et al., 2016; Kokare et al., 2017; Kyzar et al., 2017), the light–dark box (Slawecki et al., 2004; Pandey et al., 2015; Sakharkar et al., 2016; Vetreno et al., 2016; Lee et al., 2017), the marble-burying test (Lee et al., 2017), and the open-field test (Coleman et al., 2014; Vetreno et al., 2014). Together, these findings indicate that adolescent alcohol exposure as modeled by AIE causes long-lasting adult increases in alcohol drinking, risky decision-making, reward-seeking and anxiety-like behavior as well as reduced executive function that all increase risks for AUD. These adolescent alcohol-induced AUD risk factors persist long after adolescence without further alcohol exposure in adulthood. The persistence of these behaviors following AIE is consistent with a permanent change in neurobiology from adolescent alcohol exposure that increases lifelong risks for AUD that could explain the link between age of drinking onset and lifetime AUD and alcohol-related problems.

Previous reviews have covered the effect of AIE exposure on adult alcohol responses; increases in alcohol drinking, risky behaviors, and anxiety; and loss of behavioral flexibility (Spear and Swartzwelder, 2014; Spear, 2015, 2016a, 2016b, 2018; Varlinskaya and Spear, 2015; Crews et al., 2016). A recent review specifically addressed the role of sex in AIE responses (Robinson et al., 2021). This review presents new findings on the mechanisms of AIE-induced persistent changes in adult brain focusing on HMGB1, an endogenous cytokine-like molecule that can activate multiple proinflammatory receptors including most Toll-like receptors (TLR) and three interrelated examples of AIE-induced changes in adult neurobiology: (i) adult hippocampal neurogenesis, (ii) microglia, and (iii) basal forebrain cholinergic neurons. HMGB1 is released by ethanol in part in extracellular vesicles (EVs) that activate proinflammatory gene induction (Zou and Crews, 2014; Coleman et al., 2017, 2018; Crews et al., 2013, 2021b) and inhibition of HMGB1 prevents proinflammatory gene induction caused by ethanol (Crews et al., 2013, 2021b). The effect of HMGB1 proinflammatory signaling on adult hippocampal neurogenesis also affects neurocircuitry and behavior. Adult hippocampal neurogenesis provides an index of neuronal growth and plasticity that is controlled by a tightly regulated neurogenic niche environment. AIE reduces adult hippocampal neurogenesis and increases high-mobility group box 1 (HMGB1) and proinflammatory gene induction, linking loss of neurogenesis to increases in neuroimmune signaling in the neurogenic niche. Microglia, the resident innate immune cells of brain, are key regulators of neuroimmune signaling and have multiple phenotypes that influence synaptic circuits, neurons, and hippocampal neurogenesis. Emerging studies find EVs contribute to microglial signaling and regulation of the hippocampal neurogenic niche. Another effect of AIE involves the forebrain cholinergic system, which is key to arousal, decision-making, and cue-initiated behavior. Basal forebrain cholinergic neurons project to multiple brain regions, including the hippocampus, providing important anti-inflammatory feedback to microglia and regulating neurogenesis (Macht et al., 2022). Intriguingly, AIE also persistently reduces adult cholinergic neurons in parallel with increased proinflammatory signaling within cholinergic neurons. Emerging studies indicate proinflammatory signaling causes loss of cholinergic neurons through epigenetic repression of cholinergic gene expression and neuronal shrinkage that persists long into adulthood, suggesting novel mechanisms of adolescent ethanol on adult brain pathology. Interestingly, these epigenetic mechanisms led to discoveries that AIE-induced adult pathology is reversible and provide novel avenues for investigation into potential therapeutics for AUD.

## IV. Hippocampal Neurogenesis and the Neurogenic Niche: HMGB1 and Shifts in the Trophic/Proinflammatory Balance

The hippocampal subgranular zone is a unique neurogenic niche, which allows continuing new neuron birth and functional integration into local circuitry throughout adolescence and adulthood (Altman and Das, 1965; Lazic, 2012). However, the birth, differentiation, and functional integration of hippocampal newborn neurons is sensitive to disruptions in the balance between trophic support and proinflammatory signaling (Macht et al., 2020a). Ethanol disrupts this balance in the neurogenic niche, impairing neurogenesis across development (Macht et al., 2020a), but with particularly pernicious effects in adolescence. For example, in adulthood, ethanol inhibits hippocampal neurogenesis transiently but recovers during abstinence (Nixon and Crews, 2002; Crews et al., 2006; Crews and Nixon, 2009); however, adolescents have approximately fourfold more new neurons forming than adults (He and Crews, 2007; Kozareva et al., 2019), and AIE causes a persistent loss of adult hippocampal neurogenesis not observed with identical adult alcohol treatment (Broadwater et al., 2014). Further, the AIE-induced loss of neurogenesis persists for months and likely for life (Vetreno and Crews, 2015). These findings indicate that adolescent hippocampal neurogenesis is uniquely sensitive to the effects of ethanol both in magnitude and duration, producing long-term consequences on adult brain and behavior. For example, the persistent diminution of hippocampal neurogenesis by adolescent ethanol exposure is linked to adult reversal learning impairments, increased perseveration, and/or loss of cognitive flexibility, which persist at least to middle age in rodents (Vetreno and Crews, 2015; Macht et al., 2020b).

The finding that adolescent alcohol exposure increased HMGB1 proinflammatory signaling in adult brain led to studies that inhibit HMGB1 proinflammatory signaling to determine how it affects neurogenesis and loss of cognitive flexibility. Wheel running voluntary exercise and pharmacological treatments [e.g., indomethacin, galantamine, donepezil, trichostatin A (TSA)] have been found to prevent and restore the AIE-induced hippocampal loss of neurogenesis and behavioral pathology, further indicating that AIE-induced loss of neurogenesis and cognitive-behavioral impairments are linked (Sakharkar et al., 2016; Vetreno et al., 2018; Swartzwelder et al., 2019; Macht et al., 2021). While AIE increases several proinflammatory signals, evidence suggests that the innate immune factor HMGB1 is a central mediator of many AIE-induced pathogenic signaling cascades. HMGB1 and other proinflammatory genes are increased by AIE in hippocampus (Vetreno et al., 2018), whereas the trophic factor BDNF is decreased (Sakharkar et al., 2016), suggesting that AIE disrupts the proinflammatory-trophic balance in the neurogenic niche. However, AIE induction of HMGB1 signaling is blocked and reversed by treatments that can also restore the AIE-induced loss of neurogenesis and reversal learning. For example, the nonsteroidal anti-inflammatory drug indomethacin and the cholinesterase inhibitors galantamine and donepezil block the AIE-induced loss of neurogenesis and increases in hippocampal HMGB1 (Swartzwelder et al., 2019; Macht et al., 2021). Similarly, TSA, a histone deacetylase inhibitor that also exhibits anti-inflammatory properties, also restores the AIE-reduced loss of adult neurogenesis and decreased hippocampal BDNF expression, consistent with the hypothesis that AIE decreases neurogenic niche trophic signaling in conjunction with increasing niche proinflammatory signals (Sakharkar et al., 2016). TSA also reverses AIE-induced changes in amygdalar histone acetylation, increases in adult anxiety, and increases in ethanol self-administration (Pandey et al., 2015). Restoration of neurogenesis and the proinflammatory balance within the neurogenic niche also restores cognitive flexibility deficits during reversal learning on the Morris water maze (Vetreno et al., 2018). As discussed in the following text, the loss of cholinergic anti-inflammatory signals may also contribute to hippocampal loss of neurogenesis (for a review, see (Macht et al., 2020a). Understanding the mechanisms by which AIE persistently increases proinflammatory signaling throughout adulthood is key to the AIE-induced loss of hippocampal neurogenesis.

## V. AIE and HMGB1 Signaling

AIE induces a subtle but persistent increase in expression of innate immune genes in brain, including the proinflammatory signaling factors chemokine C-C motif ligand 2, cytokines TNF*α* and IL1*β*, HMGB1, and cyclooxygenase-2 as well as expression of a spectrum of innate immune signaling Toll-like receptors (TLR; i.e., TLR1, TLR2, TLR4, TLR5, TLR6, TLR7, and TLR8) and the receptor for advanced glycation end products (RAGE) (Coleman et al., 2018; Vetreno et al., 2018; Swartzwelder et al., 2019; Macht et al., 2021). An interesting property of HMGB1 and other proinflammatory signaling cytokines is the feed-forward amplification of signaling due to signaling upregulating both innate immune receptors and their activating ligands. This signaling amplification is highlighted by HMGB1. Ethanol releases HMGB1 in free form and in extracellular vesicles (EVs). In both cases, HMGB1 can form monomers as well as dimers and heteromeric complexes with other signaling molecules, functioning as a pan-proinflammatory factor activating multiple proinflammatory signaling receptor cascades (Crews et al., 2013, 2021b; Zou and Crews, 2014). HMGB1 heteromeric complexes are particularly dynamic, forming with cytokines, extracellular DNA, RNA, and damage-associated molecular pattern molecules (Yang et al., 2021). For example, HMGB1 complexes with IL1*β* magnify IL1*β* stimulation of the IL1*β*R receptor, resulting in increased expression of IL-6 and interferon-*β* (Coleman et al., 2018). HMGB1 complexes activate essentially all TLRs, making it an important proinflammatory driver (Harris et al., 2012). For example, TLR7 is activated by RNA, including endogenous microRNA (miRNA) let7 and HMGB1-let7 dimers, which are both potent agonists. Interestingly, HMGB1-let7 dimers are released together in EVs from microglia, triggering TLR7-mediated neuronal death (Coleman et al., 2017). EV contents require lysis and have only recently been discovered to contribute to TLR and cytokine receptor proinflammatory signaling cascades that converge on innate immune gene transcription regulators, like phosphorylated nuclear factor kappa light chain enhancer of activated B cells p65 (pNF*κ*B p65), which is increased in brain by AIE, consistent with induction of proinflammatory gene transcription (Vetreno et al., 2018). Collectively, these findings suggest that AIE increases HMGB1 signaling, resulting in multiple feed-forward proinflammatory signaling cascades that converge on NF*κ*B p65 to affect a wide variety of cellular networks.

In the hippocampal dentate gyrus neurogenic niche, the increase in NF*κ*B p65 activation positively correlates with activation of the executioner caspase-3 within neuroprogenitors, highlighting doublecortin+ (DCX+) neuroprogenitor proinflammatory signaling and loss of neurogenesis in the hippocampus after AIE (Vetreno et al., 2018). Activation of caspase-3 after AIE in DCX+ immature neurons suggests that HMGB1 or other proinflammatory signaling directly drives cell death of immature neurons, resulting in a persistent loss of neurogenesis (Macht et al., 2021). These findings suggest that HMGB1 and other proinflammatory signals reduce neurogenesis through increasing neuroprogenitor cell death and is a central mediator of the persistent AIE brain pathology in adulthood.

## VI. Microglia, HMGB1, and Adolescent Alcohol Exposure

**Fig. 1:**
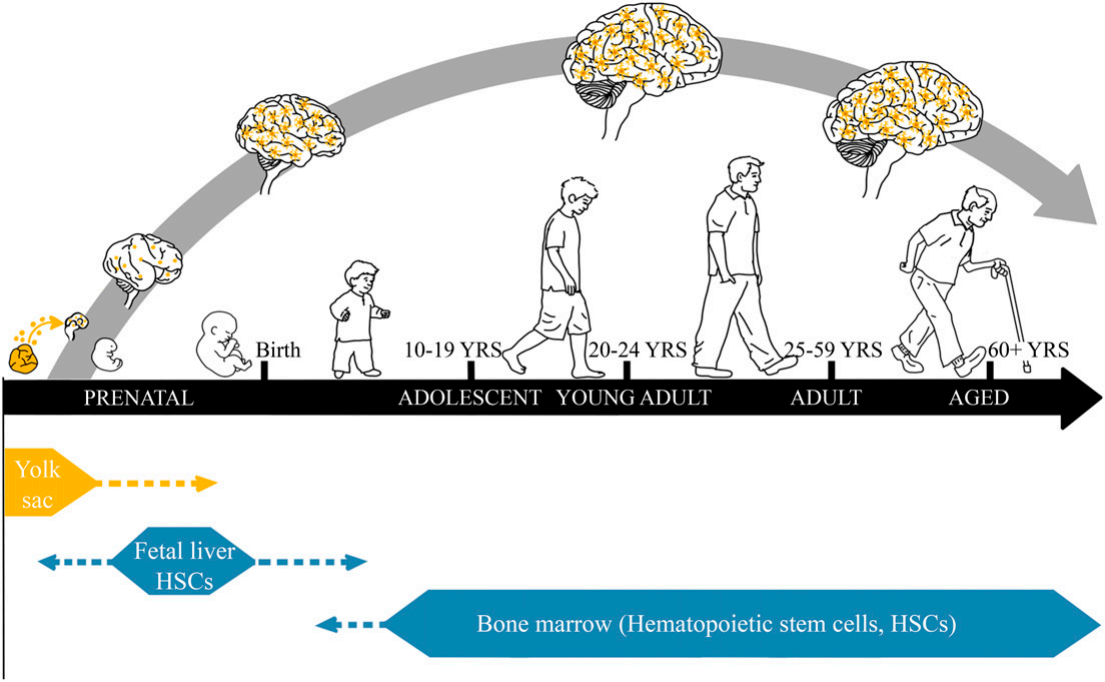
Brain development stages and microglia. Mesenchymal yolk sac progenitors migrate to neuroectodermal developing neuroprogenitors in the first trimester and become dynamically involved in brain maturation. Although poorly understood, microglia appear to play a key role supporting developing neurons and synapses as well as removing dysfunctional synapses and dying cells. Maturation to adult-like microglia parallels brain maturation, resulting in resident brain-specific innate immune monocyte-like cells, microglia. Embryonic myeloid cells enter the embryonic liver and become hepatic Kupffer monocyte-like cells. Bone marrow hematopoietic stem cells form vascular monocytes in adolescence and adulthood. Thus, microglia are brain-specific monocyte-like cells that mature with neurons, glia, and other brain cells across youth, adolescence, adulthood, and senescence. Microglia can be primed during chronic alcohol exposure, which affects brain development, neurocircuitry, and behavior.

Microglia are resident tissue-specific monocyte-like glia that are long-lived but can also divide from endogenous progenitors throughout the lifespan. These unique brain cells are formed from early embryonic yolk sac progenitors that migrate to the brain where they are dynamic contributors to brain development, maturing in adolescence to adult resident brain-specific monocyte-like glia (Fig. 1) (Brenhouse and Schwarz, 2016). Unlike other cell types in brain, which are fixed in location by the extracellular matrix, microglia are not anchored to other cells, but actively move within their brain region. Microglial density within each brain region is relatively stable and if altered, microglia proliferate to return to the “homeostatic” density, suggesting local regulatory microglial niche mechanisms (Tremblay et al., 2011).

Adolescent brain development involves maturation of synaptic circuitry, particularly interneuron regulation of circuits and myelination of important nerve tracks. Microglia, as well as astrocytes, oligodendrocytes, and neurons, contribute to maturation of adult neurocircuits. In fact, microglial maturation occurs in parallel with brain synaptic maturation, regulating axon guidance (Squarzoni et al., 2014), production of growth factors (Lenz et al., 2013; Parkhurst et al., 2013; Kopec et al., 2018; VanRyzin et al., 2018), neurite outgrowth (Pont-Lezica et al., 2014), synaptic organization (Paolicelli et al., 2011), and elimination of apoptotic neurons (Cunningham et al., 2013), among many other functions (Bordt et al., 2020). One example of the effect of microglia on brain circuit maturation is the finding that adolescent play behavior develops only in adolescent males due to sex-specific microglial elimination of striatal synaptic dopamine D1 receptors through phagocytosis that triggers male play behaviors (Kopec et al., 2018). In transgenic mice with depleted numbers of microglia, there is reduced synaptic pruning during adolescence, resulting in adults with more synapses but reduced cortical function (Wieghofer and Prinz, 2016). Similarly, the level of microglial gene expression correlates with cortical thickness during childhood and early adolescence (Vidal-Pineiro et al., 2020), and cortical thickness is linked to development of adult characteristics (Lenroot and Giedd, 2006; Bava and Tapert, 2010). Adolescent maturation also involves synaptic integration of cholinergic, noradrenergic, serotonergic, and dopaminergic cortical projection neurons into cortical–cortical and cortical–limbic circuits known to be altered by ethanol and stress (Crews et al., 2005; Casey and Jones, 2010), suggesting the ethanol-induced microglial alteration of neural circuitry may contribute to the persistence of AIE-induced molecular and behavioral phenotypes in adulthood.

Although microglia are critical for neurodevelopment during adolescence, in general, little is known about adolescent development of microglia and their role in concurrent shifts in immune system function. Maturation of the hypothalamic–pituitary stress axis across adolescence, which provides important negative feedback on immune responses, suggests there are endocrine-driven maturational changes that could affect adult microglial and other immune signaling responses (McCormick and Mathews, 2010). However, there are overt changes in microglia across adolescence that likely also influence microglial role in immune function: the number of microglia increases in mice, with females showing a greater increase (Schwarz et al., 2012). The transcriptional profiles of microglia also change with development. In human brain, expression of the prophagocytic marker CD68 increases across adolescence (Mildner et al., 2017), and the expression of several TLRs in brain change during adolescence from early development to adulthood (Kaul et al., 2012). This includes TLR4 and TLR7, both of which are involved in neuroimmune pathology associated with ethanol (Montesinos et al., 2016a; Qin et al., 2021). These changes suggest that adolescence may represent a critical period for maturation of microglia, but whether adolescent microglia are more sensitive to immune stimuli such as binge drinking remains an open question.

### A. HMGB1 Priming of Microglia and Extracellular Vesicles

Microglia play a key role in initiating proinflammatory signaling in response to binge levels of ethanol (Coleman et al., 2017; Walter and Crews, 2017; Warden et al., 2021). An important feature of microglia that is relevant to adolescent alcohol exposure is their ability to become “primed” by immune stimuli. Priming means exposure to an immune stimulus increases future responsiveness to that or similar stimuli. Microglia can be primed by stressors or proinflammatory insults such as TLR agonists (Niraula et al., 2017; Neher and Cunningham, 2019). Evidence supports that adolescent binge ethanol exposure increases microglial reactivity to future stressors in adulthood (Walter et al., 2017). One study of adolescent binge ethanol exposure found disruption of novel object learning and hippocampal long-term synaptic depression are blocked by minocycline, which inhibits microglial activation by ethanol, as well as by the TLR4 antagonist TAK-242, which blocks HMGB1, and the anti-inflammatory drug indomethacin (Deschamps et al., 2022), which has multiple anti-inflammatory mechanisms, further linking ethanol to microglial activation. Another AIE study found increased pain sensitivity in adults that was alleviated by minocycline (Khan et al., 2022). These studies support AIE priming of microglia, although stress can also prime microglia (Walter et al., 2017). Chronic adolescent stress, for instance, results in increased microglia responsive to lipopolysaccharide (LPS) in adulthood (Bekhbat et al., 2021). Additional studies are needed to clearly understand the detailed mechanisms of microglial priming by ethanol.

Ethanol promotes microglial activation through the release of endogenous damage-associated molecular patterns such as the endogenous TLR regulator HMGB1 (Crews et al., 2013, 2021b; Zou and Crews, 2014; Coleman et al., 2017, 2018). HMGB1 is a multifaceted immune regulator that translocates from the nucleus to the cytoplasm and is ultimately released in response to ethanol. HMGB1 can be secreted from both neurons and microglia (Zou and Crews, 2014; Coleman et al., 2017), and neutralization of HMGB1 prevents proinflammatory gene induction caused by ethanol (Crews et al., 2013, 2021b). Recently, neuronal HMGB1 has been implicated as a driver of microglial activation in peripheral pain models (Yang et al., 2021), highlighting that HMGB1-microglial signaling may play important roles in the pathophysiology of diseases involving innate immune dysfunction.

Our understanding of HMGB1 signaling continues to expand as evidence now suggests that HMGB1 is also secreted in EVs that are capable of regulating function of recipient cells through delivery of diverse cargo (Pisetsky, 2014; Chen et al., 2016; Coleman et al., 2017; Crews et al., 2021b). EVs are released from cells primarily as exosomes (40–100 nm diameter) and microvesicles (0.1–1.0 *µ*m diameter), and transfer protein, nucleic acid, and lipid cargo (Jeppesen et al., 2019). EVs have been implicated as drivers of proinflammatory signaling in peripheral and central nervous system pathologies (Buzas et al., 2014; van Niel et al., 2018; Vassileff et al., 2020), including ethanol (Coleman et al., 2017; Ibanez et al., 2019, 2021; Crews et al., 2021b). In fact, we found that blocking EV release in brain slice cultures abolished proinflammatory gene induction in response to ethanol (Crews et al., 2021b).

**Fig. 2:**
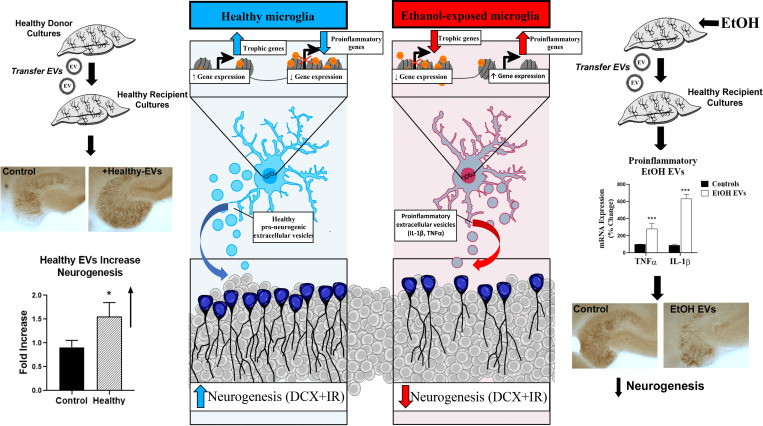
Microglial secretions regulate hippocampal neurogenesis. Emerging evidence supports microglial signaling through EVs. Generally free cytokines as followed, but not EV signaling. In vivo, AIE increases adult HMGB1 and proinflammatory genes as well as reducing adult hippocampal neurogenesis (DCX+IR). In ex vivo brain slice cultures, EVs can be isolated from the media, washed, and reapplied to naïve cultures (see Zou et al., 2022). This study found EVs from healthy control slices stimulated hippocampal slice culture neurogenesis, whereas EVs from ethanol exposure cultures reduced neurogenesis and increased proinflammatory cytokines. Far left: Ex vivo studies find microglia release healthy EVs from control cultured brain slices that can be purified and increase neurogenesis (DCX+IR) in recipient slices. This supports healthy microglia as promoting neurogenesis and growth. Far right: Ethanol-treated hippocampal slices release EVs that stimulate formation of TNF*α*-IL-1*β* and decrease neurogenesis (DCX+IR), consistent with a change in microglial phenotype reflected in ethanol-induced changes in EV signaling. Middle: Schematic of microglial phenotype shifts from release of healthy EVs (blue) to proinflammatory EVs (red) that regulate neurogenesis. Additional studies are needed to better understand shifts in microglial signaling, innate immune memory and microglial priming.

As previously discussed, AIE persistently impairs hippocampal neurogenesis in vivo (Broadwater et al., 2014; Vetreno et al., 2018; Macht et al., 2020a, 2020b). We recently observed that EVs from healthy brain culture slices were trophic and promoted neurogenesis (Fig. 2, left panel). However, EVs from ethanol-treated slice cultures not only cause proinflammatory gene induction but also a loss of adult hippocampal DCX+ neurogenesis similar to that caused by ethanol (Zou et al., 2022) (Fig. 2, right panel). This inhibition of DCX+ cells involves increased dimethylation of histone 3 lysine 9 (H3K9) residues by the euchromatic histone methyltransferase 2 (G9a) as G9a inhibitors prevent the loss of neurogenesis caused by ethanol and proinflammatory ethanol-induced EVs (Zou et al., 2022). This suggests that proinflammatory activation of microglia may lead to epigenetic modifications in neurons to produce neuropathology through the secretion of EVs. Also, microglial proinflammatory priming may also be regulated by epigenetic modifications in response to ethanol. In other settings, epigenetic histone chromatin modifications can promote proinflammatory microglial priming (Cheray and Joseph, 2018). Thus, interventions that prevent EV signaling and/or restore epigenetic modifications caused by ethanol could prevent long-term deficits cause by adolescent binge drinking.

## VII. AIE Causes a Persistent Loss of Forebrain Cholinergic Neurons

Forebrain cholinergic neurons form an extensive network of vast projections to multiple brain regions, including the cortex, hippocampus, and amygdala. Cholinergic neurons modulate cortical arousal affecting cognitive and emotive function and integrating neural networks (Vandecasteele et al., 2014; Li et al., 2018). Multiple studies have found that AIE reduces immunohistological expression of the acetylcholine synthesizing enzyme choline acetyltransferase (ChAT) in the medial basal forebrain (Coleman et al., 2011; Ehlers et al., 2011; Vetreno et al., 2014; Swartzwelder et al., 2015; Fernandez and Savage, 2017). Indeed, ChAT+IR neurons are reduced approximately 30% just after AIE at P56, a reduction that persists well into adulthood until P220, perhaps for the duration of life (Coleman et al., 2011; Ehlers et al., 2011; Vetreno et al., 2014; Swartzwelder et al., 2015; Fernandez and Savage, 2017). In addition, the remaining ChAT+IR cholinergic neurons are smaller, consistent with somal shrinkage (Vetreno and Crews, 2018; Vetreno et al., 2020; Crews et al., 2021a). Studies find immunohistochemical and mRNA reductions of ChAT as well as the vesicular acetylcholine transporter (VAChT), and the high- and low-affinity nerve growth factor receptors TrkA and NGFR (Vetreno and Crews, 2018; Vetreno et al., 2020;Crews et al., 2021a), which are highly expressed markers of cholinergic neurons (Vetreno and Crews, 2018; Vetreno et al., 2020) and important for cholinergic neuron development (Isaev et al., 2017). The AIE-induced loss of ChAT is specific to AIE exposure as identical intermittent ethanol treatment in adulthood does not affect forebrain cholinergic neurons (Vetreno et al., 2014). Intriguingly, postmortem human basal forebrain samples from individuals with AUD and an adolescent age of drinking onset show reductions of ChAT and VAChT similar to AIE-treated rats (Vetreno et al., 2014). The AIE-induced loss of basal forebrain cholinergic neurons is accompanied by loss of cholinergic receptors (Coleman et al., 2011) as well as diminished ACh frontal cortical efflux during maze performance (e.g., prefrontal cortex) (Fernandez and Savage, 2017). However, parvalbumin GABAergic neurons in the basal forebrain are not reduced by AIE (Coleman et.al. 2011), highlighting the selective increased vulnerability of adolescent ChAT+IR cholinergic neurons to binge alcohol exposure. Thus, adolescent, but not adult, AIE binge ethanol exposure decreases basal forebrain cholinergic neuron populations and causes somal shrinkage of the residual ChAT+ cholinergic neurons that persists into adulthood, which is likely to contribute to lasting adult cognitive deficits even in the absence of additional alcohol consumption.

### A. Forebrain Cholinergic Neurons, Epigenetic Silencing, and Reversal

Similar to the molecular mechanisms underlying loss of hippocampal neurogenesis, AIE-induced loss of forebrain cholinergic neurons is also linked to increases of HMGB1 and TLRs. In fact, anti-inflammatory interventions, including indomethacin, exercise, and galantamine treatments during AIE block the loss of ChAT+ neurons (Vetreno and Crews, 2018; Vetreno et al., 2020;Crews et al., 2021a). In contrast, treatment with the TLR4 agonist LPS during adolescence mimics the AIE-induced loss of ChAT (Vetreno et al., 2014; Vetreno and Crews, 2018), further emphasizing that neuroimmune induction is a key component underlying loss of ChAT+ cholinergic neurons. AIE increases forebrain expression of TLR4 and RAGE receptors, HMGB1, and the nuclear transcription factor pNF*κ*B p65 signal known to be activated in response to HMGB1/TLR4 signaling (Vetreno and Crews, 2018; Crews et al., 2021a). Our studies find that voluntary wheel running, exercise, and the anti-inflammatory drug indomethacin, administered during AIE prevent HMGB1-TLR4/RAGE-pNF*κ*B p65+IR within ChAT+IR neurons as well as loss and shrinkage of ChAT+ cholinergic neurons (Vetreno and Crews 2018). The persistent AIE-induced loss of basal forebrain cholinergic neurons was originally interpreted as cell death since AIE decreases expression of cholinergic neuron markers, including ChAT, VAChT, and the receptors for nerve growth factor, TrkA, and NGFR, as well as cholinergic neuron lineage genes. While all of these markers are significantly reduced by AIE, there was no clear evidence of neuronal death, prompting experiments to determine whether the loss of ChAT+ neurons was due to epigenetic gene silencing of cholinergic genes.

Epigenetic repression as an underlying mechanism of ChAT phenotypic suppression opens exciting new possibilities with treatments aimed at reversal of AIE pathology. We assessed whether anti-inflammatory wheel exercise, started after cessation of ethanol exposure, and the AIE-induced loss of ChAT+ neurons could restore basal forebrain cholinergic neurons. Surprisingly, exercise reversed the AIE induction of HMGB1-TLR4/RAGE-pNF*κ*B p65 neuroimmune signaling and the loss of ChAT+, TrkA+, and NGFR+ cholinergic neurons as well as somal shrinkage. We found no changes in total basal forebrain NeuN+ neuron numbers in the adult AIE-treated basal forebrain that had lost ChAT+ neurons and no BrdU+ neurogenesis (Vetreno et al., 2020; Crews et al., 2021a). Anti-inflammatory treatments following AIE, including exercise and galantamine, that restore ChAT+ neurons support the hypothesis of reversible epigenetic repression of cholinergic genes.

**Fig. 3:**
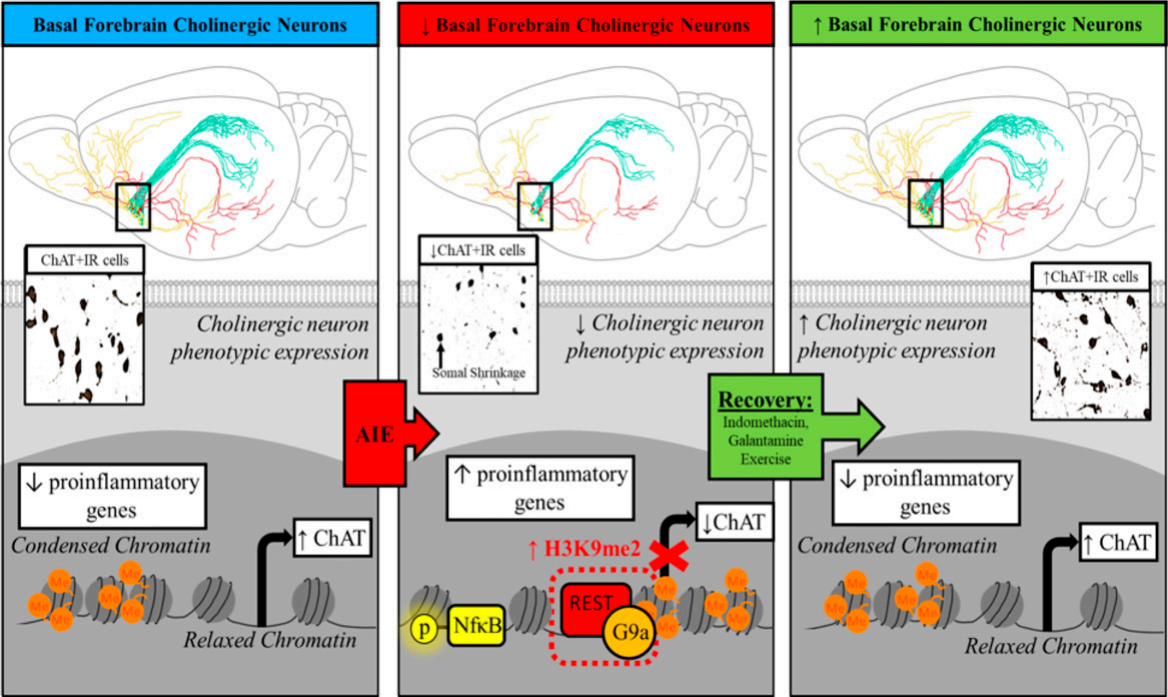
Epigenetic cholinergic gene silencing and shrinkage of cholinergic neurons of cholinergic neurons following AIE. In vivo AIE reduces cholinergic neurons. There is a loss of ChAT and multiple other cholinergic neuron genes, but no loss of neurons. Studies on prevention and restoration of cholinergic neurons using anti-inflammatory treatments support loss of the differentiated cholinergic neuron phenotype and not neuronal death (Vetreno et al., 2020). As illustrated, AIE increases gene silencing through induction of REST that recruits G9a, a histone methylating enzyme that silences transcription of cholinergic genes. Top: Schematic of rat brain cholinergic neuron projections (green, red, yellow) across multiple brain regions. Middle: Immunohistochemistry of choline acetyltransferase (ChAT+IR neurons) cholinergic neurons in basal forebrain. Note that in the left and right panel ChAT+IR neurons are larger, while in the middle panel AIE decreases ChAT+IR cholinergic neurons and causes somal shrinkage of the remaining ChAT+ neurons. Bottom: Schematic of relaxed open chromatin transcribing ChAT and condensed chromatin or NF*κ*B regulating transcription of proinflammatory genes. The left panel shows normal control ChAT transcription and condensed chromatin reflecting no proinflammatory gene transcription. The middle panel schematic of AIE illustrates loss of ChAT+ neurons and projection loss (top middle panel), somal shrinkage (ChAT+ images middle), and silencing of ChAT expression through REST-G9a epigenetic mechanisms (bottom middle panel). Proinflammatory transcription factor NF*κ*B is increased in ChAT+ cells consistent with increased proinflammatory gene induction. AIE-induced adult ChAT loss is reversible (right panel) with treatments blocking HMGB1 proinflammatory signals and gene silencing REST-G9a and restoration of ChAT+ cell number, somal size, and projections.

Collectively, these findings emphasize that adolescent binge ethanol exposure and neuroimmune induction can elicit long-lasting epigenetic changes in cellular programming through changes in DNA and nuclear histone modifications that enhance or suppress gene expression (Montesinos et al., 2016b; Pandey et al., 2017; Wolstenholme et al., 2017; Vetreno et al., 2020; Crews et al., 2021a). Of particular relevance, *Chat* and other cholinergic genes contain the consensus 21-base-pair DNA binding sequence REl (also known as neuron-restrictive silencer element) that binds the transcriptional repressor RE1-silencing transcription factor (REST; also known as neuron-restrictive silencer factor) (Shimojo and Hersh, 2004; Abrajano et al., 2009). REST is known to regulate cholinergic gene expression during development (Shimojo and Hersh, 2004). REST recruits the methyltransferase G9a, which silences gene transcription through H3K9 dimethylation (Roopra et al., 2004; Ballas et al., 2005), and methylation of H3K9 can repress gene transcription (Wang et al., 2008). AIE increased H3K9 dimethylation occupancy at gene promoter regions of *Chat* and *Trka* as well as DNA methylation at the CpG island of the *Chat* gene promoter (Vetreno et al., 2020; Crews et al., 2021a), consistent with reduced gene expression. Taken together, these findings suggest HMGB1-TLR4/RAGE signaling activates pNF*κ*B p65 neuroimmune signaling within cholinergic neurons, increasing expression of REST-G9a leading to H3K9 dimethylation suppression of cholinergic phenotype genes, resulting in a loss of some neurons and shrinkage of others (Fig. 3). Although the AIE ChAT+ neuron loss is persistent, we never find loss of more than 30% to 40%, suggesting a limit to the efficacy of epigenetic silencing. Although poorly understood, neuroimmune-linked epigenetic repression of neuronal phenotype represents a previously unappreciated mechanism of neuronal plasticity. Restoration of the AIE-induced persistent loss of basal forebrain cholinergic neurons through reversal of persistent neuroimmune-induced changes in chromatin epigenetic signaling may offer great promise for reversing AIE pathology as well as other pathologies initially thought to be neurodegeneration.

## VIII. Reversal of AIE Pathology Identifies New Mechanisms and Potential Therapies

The finding that AUD cortical and hippocampal brain regions have increased expression of HMGB1, TLR receptors, and other proinflammatory molecules overlaps with other mental and neurodegeneration-related diseases, many of which are comorbid with AUD. Not surprisingly, there is an increasing number of preclinical and clinical trials under way investigating anti-inflammatory immune therapies for AUD. Anti-inflammatory targets discussed next include HMGB1 and TLR receptors, phosphodiesterase (PDE) inhibitors; inhibition of microglial proinflammatory priming, anti-inflammatory acetylcholine/*α*7 nicotinic agonists, and modifiers of histone methylation-acetylation.

### A. Inhibition of HMGB1/TLR Signaling and Phosphodiesterases

**Fig. 4:**
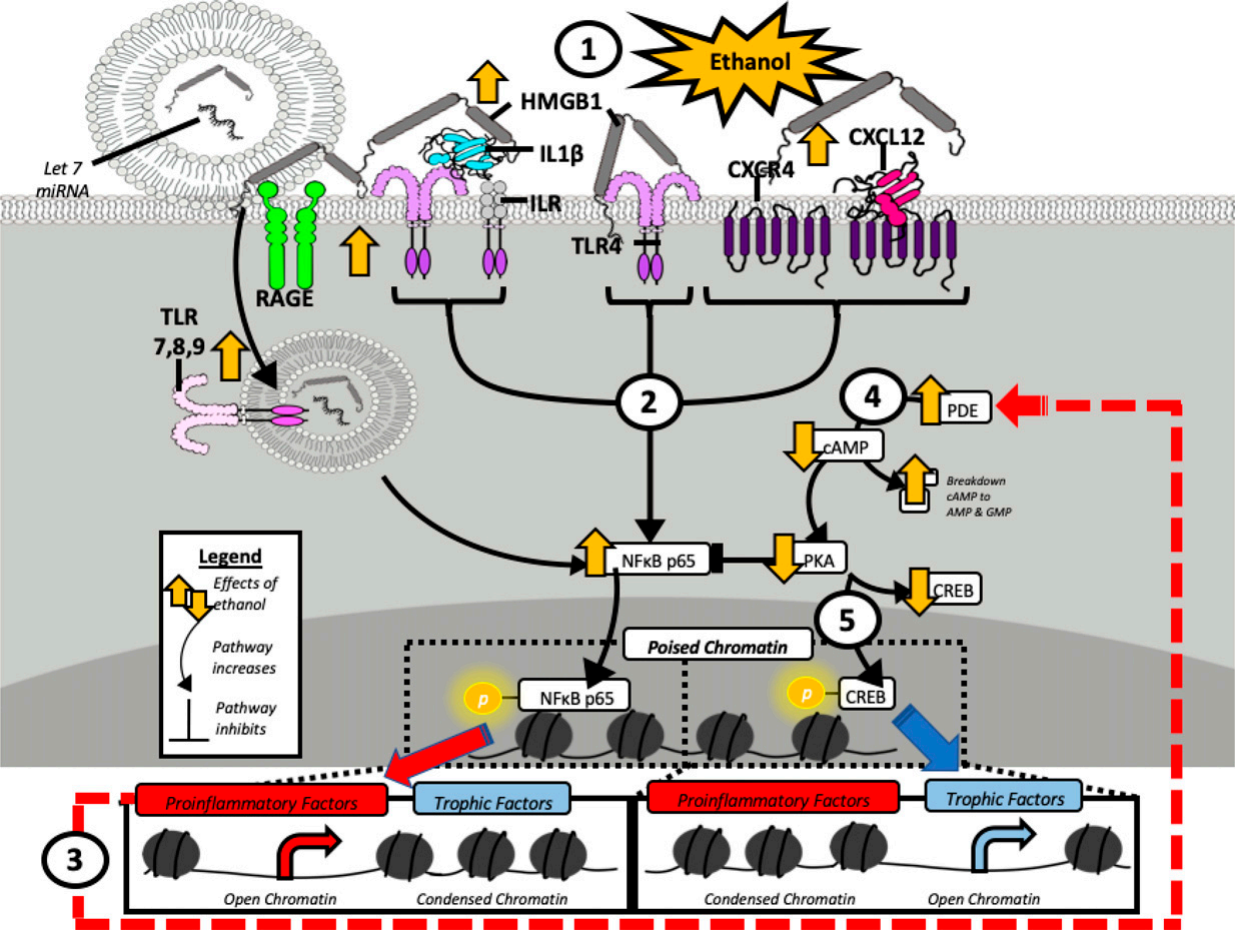
Schematic of neuroimmune targets for treatment of AUD. Several targets are identified from studies of AIE adult neuropathology. Target 1 is HMGB1, and Target 2 is the multiple TLR and other receptors as well as their common NF*κ*B signaling. Target 3 is the epigenetic mechanisms increasing proinflammatory genes and reducing trophic factor and neurotransmitter gene expression. Target 4 is PDE that shifts NF*κ*B/cAMP signaling. Target 5 is agents increasing trophic factor transcription. Target 1 is HMGB1. Acute ethanol releases HMGB1, activating signaling cascades of innate immune genes. HMGB1 and TLR receptors are induced in brain by chronic ethanol exposure, sensitizing multiple TLR responses (Qin and Crews, 2012; Qin et al., 2013, 2021). HMGB1 can activate multiple Toll-like receptors and other innate immune receptors through the formation of heteromers. It is poorly understood which brain cells release HMGB1 in response to ethanol and other immune activators. For example, the HMGB1 antagonist glycyrrhizin, as well as HMGB1-neutralizing antibodies, block both glutamate and TNF*α*-induced neuronal death in brain slice cultures (Zou, 2015). Further, EVs containing HMGB1 as well as free HMGB1 are released, and how this release and the diverse receptor responses interact is not understood. For example, HMGB1 is shown as a gray molecule to two thick arms representing binding boxes. HMGB1 activates multiple innate immune receptors as shown, some through heteromer-increased potency of other agonists. Examples of HMGB1 signaling include (left) RAGE receptor and HMGB1-Let7 containing EVs (Coleman et al., 2017a), (middle) HMGB1-IL1*β* heteromers activating IL1R (Coleman et al., 2018), HMGB1 alone activating TLR4 or RAGE, and HMGB1-CXCL12 heteromers activating G protein coupled CXCR4 receptors (Harris et al., 2012). These receptors are each targets (Target 2) and are the epigenetic enzymes altering transcriptional balance (Target 3) as well as PDE induction (Target 4) that decreases cAMP-BDNF transcription (Target 5) and protein kinase A blunting of NF*κ*B transcription increasing proinflammatory genes (Avila et al., 2017). These AIE-identified targets may have indications broader than AUD since many psychiatric and neurologic disorders are also associated with increased neuroimmune gene expression.

TLRs are part of the TLR/IL1 receptor superfamily that activate NF*κ*B transcription of proinflammatory, innate immune, and many other genes. NF*κ*B has many target genes but is most commonly linked to proinflammatory gene induction. TLR and other proinflammatory signaling regulate immune cell phenotype in part through formation of polychrome complexes that recruit histone-modifying polychrome or other complexes that enhance or repress specific genes. Microglia are well known to have multiple phenotypes that change during development and with drug treatment. The findings that exercise (running wheel), indomethacin (a direct anti-inflammatory drug), and galantamine and donepezil (anti-cholinesterase drugs) block the AIE-induced increases in proinflammatory HMGB1-TLR-RAGE signaling and loss of neurogenesis and cholinergic neurons as well as cognitive deficits, supporting HMGB1-TLR-RAGE signaling as a mechanism of AIE-induced AUD-like pathology. AUD is treated with opiate antagonists, and both human and preclinical studies support an off-target TLR4 blockade by opiate antagonists naltrexone, naloxone, and nalmefene as the mechanism of reduced heavy drinking in human AUD trials using these drugs (Meredith et al., 2021). There are also ongoing AUD clinical trials with neuroactive steroids based on preclinical reductions in ethanol drinking, preference, and operant responding due to blockade of TLR receptors, particularly TLR4 and TLR7 (Morrow et al., 2020; Balan et al., 2021). Ethanol exposure induces and releases HMGB1, primarily from neurons (Zou and Crews, 2014), which triggers microglial HMGB1-EV signals to adjacent astrocytes, other glia, and neuroprogenitors, spreading a proinflammatory milieu. HMGB1 is concentrated in the nucleus of neurons and other cells. In hippocampal brain slice cultures, ethanol and inhibitors of histone deacetylases release acetyl-HMGB1 into the media. HMGB1+IHC and cellular fractionation find increased neuronal cytoplasmic HMGB1 and media HMGB1, consistent with neuronal release. Ethanol and exogenous HMGB1 treatment increase proinflammatory cytokines TNF*α* and IL-1*β* as well as TLR4 mRNA expression. The HMGB1 inhibitor glycyrrhizin, small interfering RNAs (siRNAs) to HMGB1 and TLR4, the HMGB1 neutralizing antibody naltrexone, and the TLR4 antagonist LPS-Rs, as well as minocycline, the microglial activation inhibitor, blocked both ethanol and exogenous HMGB1 proinflammatory gene induction (Zou and Crews, 2014). Endotoxin and LPS, the TLR4 agonist, reduce ChAT+ neurons similar to ethanol in forebrain slice cultures, and glycyrrhizin blunts responses (Crews and Vetreno, 2022). Interestingly, glycyrrhizin also blocks ethanol proinflammatory EVs released by microglia (Crews et al., 2021b). These findings support HMGB1 as a potential target to prevent and reverse ethanol-induced pathology (Fig. 4). However, these signaling mechanisms and the contributions of different cell types are poorly understood, and much more research is needed to understand the therapeutic potential of brain HMGB1 and other anti-inflammatory therapies for AUD.

HMGB1 is a particularly good target to reduce proinflammatory signaling since it has broad signaling activity across TLR, cytokine, chemokine, and other receptors. Systemic HMGB1 is known to contribute to the severity of sepsis (Zhu et al., 2021); however, there are fewer studies in the brain. HMGB1 is released from cells by both active secretion and inflammatory cell death. Both acute and chronic ethanol increase HMGB1 in blood and brain (Coleman et al., 2018; Vannier et al., 2021). HMGB1 activates a broad array of innate immune receptors. Oxidized disulfide HMGB1 is a direct TLR4 and RAGE agonist, whereas reduced HMGB1 forms heterocomplexes with agonists at TLR/IL-1 receptor (TLR/IL-1R) superfamily members as well as G protein-coupled chemokine CXCL12-CXCR4 receptors (Fig. 4). For example, in brain slice cultures, treatment with HMGB1/IL1*β* heterocomplexes formed in vitro increases TNF*α* mRNA about two- to threefold more than equivalent IL1*β* alone (Coleman et al., 2018). AUD hippocampus has increased levels of HMGB1 heterocomplexes with IL1*β* and miRNA-let7 (Coleman et al., 2017). HMGB1/IL1*β* and HMGB1/Let7miRNA heterocomplexes both are released into the media by acute ethanol treatment of rat hippocampal brain slice cultures that includes free and EV components. Ethanol increases EV with HMGB1/Let7 heterocomplexes that stimulate and induce TLR7. Thus, HMGB1 signals across multiple innate immune receptors, although mechanisms are not completely understood. Ethanol releases HMGB1 into the media of brain slice cultures as well as inducing TNFα and IL1B mRNA. Anti-HMGB1 antibodies, TLR4 siRNA, HMGB1 siRNA, and glycyrrhizin, a natural HMGB1 antagonist, block ethanol induction of TNFα and IL1B mRNA (Zou and Crews, 2014), consistent with HMGB1 being a key proinflammatory brain signal. Further, ethanol withdrawal and TLR7-sensitized toxicity are blocked by glycyrrhizin, supporting HMGB1 heterocomplexes as a target (Coleman et al., 2017). The ability of HMGB1 to activate multiple innate immune receptors supports HMGB1 as a key target to block, and glycyrrhizin analogs may have promise as anti-inflammatory and AUD therapies (Fig. 4).

### B. Phosphodiesterase Inhibitors

PDEs regulate the intracellular levels of cAMP and cGMP (Wen et al., 2018). PDEs have multiple subtypes differentially distributed in the brain, regulating cAMP and cGMP signaling pathways that play a key role in neural functions and synaptic transmission in the central nervous system as well as the downregulation of NF*κ*B and proinflammatory signaling (Parry and Mackman, 1997; Wen et al., 2018). PDEs modulate the cAMP protein kinase A pathway, which has been implicated in the regulation of response to acute and chronic alcohol exposure (Logrip, 2015). Brain slice cultures exposed to increasing concentrations of ethanol show progressive decreases in CREB-DNA binding with concentration-dependent increases in NF*κ*B–DNA binding (Zou and Crews, 2006). Rolipram, a PDE inhibitor, increased CREB–DNA binding and transcription of BDNF, reversing ethanol, NF*κ*B–DNA binding, TNF*α* induction, and increased glutamate-induced neuronal cell death (Zou and Crews, 2006). These studies support an ethanol-induced shift in transcription from CREB-trophic BDNF to NF*κ*B proinflammatory genes (Fig. 4). Other studies in mice find rolipram reduces ethanol drinking (Blednov et al., 2014). Mice fed chronic ethanol-containing diets have reduced cAMP, increased PDE4b and cytokine expression as well as activated microglia and astrocytes. Ethanol-induced increases in proinflammatory genes include induction of PDEs, particularly PDE4. Similar to the results of Blednov et al. (2014), ethanol induction of brain proinflammatory genes is reduced by the PDE4 inhibitor rolipram and in transgenic mice lacking PDE4b (Avila et al., 2017). In humans, the anti-inflammatory PDE inhibitor ibudilast has shown promise in human laboratory studies. One trial with ibudilast found decreased alcohol cue craving and improved mood in nontreatment-seeking individuals with AUD (Ray et al., 2017). In another trial using functional magnetic resonance imaging, ibudilast treatment of 2 weeks reduced heavy drinking and decreased functional magnetic resonance imaging ventral striatal reward activation responses to alcohol cues (Grodin et al., 2021). PDE inhibitors and TLR antagonists might benefit from being used in combination since they have different primary targets that reduce NF*κ*B signaling across diverse cell phenotypes (Fig. 4).

### C. Microglia

Microglial priming refers to sensitization of microglia to activation. Chronic ethanol exposure of mice increases TLR expression in brain and sensitizes brain TNF*α* mRNA induction by systemic LPS-TLR4 (Qin et al., 2013) and PolyI:C-TLR3 (Qin and Crews, 2012). Cycles of alcohol induce innate immune memory processes increasing TLR expression in brain, priming microglia and other cells increasing proinflammatory responses (Coleman et al., 2020; Meredith et al., 2021; Zhang et al., 2022). Minocycline, a broad-spectrum antibiotic that blocks microglial activation, and TNF*α* gene induction by endotoxin reduce alcohol intake in a mouse free-choice voluntary drinking model (Agrawal et al., 2011). Minocycline has also been found to reduce alcohol sedation as well as withdrawal-related anxiety and alcohol reinstatement (Wu et al., 2011; Gajbhiye et al., 2018). One study of adolescent binge ethanol exposure found disruption of novel object learning and hippocampal long-term synaptic depression is blocked by minocycline as well as the TLR4 antagonist TAK-242, which blocks HMGB1 and indomethacin (Deschamps et al., 2022), further linking ethanol to microglial neuroimmune activation. Another AIE study found increased pain sensitivity in adults that was alleviated by minocycline (Khan et al., 2022). These studies suggest microglia and TLR are involved in multiple chronic effects of ethanol exposure in adolescents and adults.

A novel microglial strategy has emerged using inhibitors to the colony-stimulated factor 1 receptor that lead to depletion of microglia, which require colony-stimulated factor 1 agonists for survival (Barnett et al., 2021). For example, administration of PLX5622 to mice results in the loss of almost all Iba1+ microglia (Walter and Crews, 2017). Microglial depletion markedly reduces TNF*α* and blunts acute ethanol induction of TNF*α*, but IL1*β* is not reduced and is enriched in astrocytes. Microglial depletion blunted ethanol-induced proinflammatory (e.g., TNF*α*, chemokine C-C motif ligand 2) gene expression and enhanced anti-inflammatory (e.g., IL-1ra, IL-4) gene expression during acute binge ethanol withdrawal (Walter and Crews, 2017). Ex vivo studies in rat brain slice cultures find that ethanol exposure, which primes microglia-inducing proinflammatory cytokines, is lost with PLX depletion of microglia. Upon removal of colony-stimulating factor 1 receptor antagonists, microglia repopulate to a more tropic, anti-inflammatory phenotype that is protective after chronic ethanol and in other neuroimmune disorders (Barnett et al., 2021; Shi et al., 2022). Microglial repopulation is effective at reducing persistent proinflammatory microglial activation caused by binge levels of ethanol (Coleman et al., 2020). Thus, microglial depletion eliminates ethanol-primed phenotypes that repopulation restores with a naïve trophic, anti-inflammatory microglial phenotype. Preclinical studies in mice do not show obvious toxicity, suggesting depletion and repopulation of microglia as an approach to reducing brain proinflammatory signaling should be further investigated.

### D. Anti-Inflammatory Anti-Cholinesterases and Acetylcholine

The anti-inflammatory actions of acetylcholine are well known from systemic and central nervous system studies (Cox et al., 2020; Zorbaz et al., 2022). Immune cells and neurons both release acetylcholine signaling through cholinergic receptors. Acetylcholine blocks LPS-elicited hippocampal toxicity and proinflammatory cytokine induction by inhibiting the microglial NF*κ*B transcription and promoting microglial neurotrophic factor secretion via *α*7 nicotinic acetylcholine receptors (*α*7nAChR) on microglia (Wang et al., 2004; Li et al., 2019). The anti-cholinesterases donepezil and galantamine increase acetylcholine by inhibiting its metabolism and reverse AIE-induced adult HMGB1-RAGE and proinflammatory gene induction in hippocampus as well as reversing the persistent loss of neurogenesis (Macht et al., 2020a). Galantamine is also a direct *α*7nAChR nicotinic receptor agonist that may enhance anti-inflammatory activity. Interestingly, varenicline, a smoking cessation medication with partial nicotinic *α*4*β*2 agonist activity, has full *α*7nAChR agonist activity and has shown efficacy in reducing craving for alcohol and heavy drinking in patients with AUD (Erwin and Slaton, 2014). Additional studies are needed to determine the potential of cholinergic and/or direct *α*7nAChR nicotinic agonists as anti-inflammatory treatments.

## IX. Summary and Conclusions

Adolescent alcohol drinking is common, and studies of early adolescent age of drinking onset or initiation universally agree that early adolescent drinking increases adolescent and lifelong alcohol drinking and alcohol-related problems, as well as risks for AUD. Multiple complex risk factors for AUD include altered reward-seeking, impulse inhibition, risky decision-making, and perseveration involving complex neurocircuits. Proinflammatory signaling including HMGB1/TLR and other innate immune genes are increased in multiple brain regions that contribute to the development of AUD (Crews et al., 2011, 2017; Cui et al., 2014; Erickson et al., 2019; Coleman et al., 2021).

Preclinical studies of adolescent alcohol exposure such as the NADIA AIE model find unique and persistent changes in neurobiology and behavior lasting into adulthood and often not found with comparable adult exposure. For example, AIE causes long-lasting adult increases in alcohol drinking, risky decisions, reward-seeking, and anxiety as well as reduced executive functions that are paralleled by increased HMGB1-TLR brain neuroimmune signaling (Crews et al., 2011, 2017). HMGB1-TLR4/RAGE signaling primes microglia, increasing proinflammatory responses. HMGB1 bound agonist heterocomplexes signal across TLR/IL1R receptors, across CXCR4 G protein chemokine receptors as free molecules, and within EVs, all of which activate NF*κ*B transcription. Each of these HMGB1 signals is a drug target for AUD. PDE inhibitors also show promise as a treatment of AUD due to PDE inhibition blocking NF*κ*B while enhancing CREB transcription, reversing proinflammatory phenotypes. Loss of hippocampal neurogenesis is related to changes in proliferation, maturation, and survival of progenitors that are altered by a proinflammatory shift in the neurogenic milieu. AIE causes a loss and shrinkage of forebrain cholinergic neurons due to increased REST repressing cholinergic genes. Treatments that restore the fully differentiated cholinergic phenotype may benefit AUD and many other brain pathologies. The brain regional changes in phenotypes related to innate immune and trophic factor gene expression induced by adolescent alcohol exposure are known to persist long after exposure has ended but are poorly understood and need additional study. Multiple new targets, including inhibitors of HMGB1 and other innate immune receptors, need to be explored to extend the anti-inflammatory indomethacin, acetylcholinesterase inhibitors, and histone deacetylase inhibitors shown to be potential therapeutic targets for AUD. Further, these mechanisms overlap with neurodegenerative disorders and may be targets for neurodegeneration diseases as well. Studies identifying EVs as contributors to innate immune signaling indicate our understanding of signals is incomplete since EV signaling is poorly understood and may mediate components of responses to ethanol. The reversal of loss of basal forebrain cholinergic neurons suggests previous studies of cholinergic degeneration need to be reassessed. The persistence of epigenetic changes in gene expression supports the hypothesis that adolescent drinking, independent of adult drinking, can increase risks for adult heavy drinking and AUD. The discovery that anti-inflammatory/epigenetic drugs can reverse AIE-induced changes at the molecular and behavioral levels provides promise for new therapies.

## Abbreviations

*α*7nAChR: alpha7 nicotine acetylcholine receptor
AIE: adolescent intermittent ethanol
AUD: alcohol use disorder
ChAT: choline acetyltransferase
DCX: doublecortin
EV: extracellular vesicle
H3K9: histone 3 lysine 9
HMGB1: high-mobility group box 1
LPS: lipopolysaccharide
miRNA: microRNA
NADIA: Neurobiology of Adolescent Drinking in Adulthood
P: postnatal day
pNF*κ*B: phosphorylated nuclear factor kappa light chain enhancer of activated B cells
PDE: phosphodiesterase
RAGE: receptor for advanced glycation end products
REST: RE-1 silencing transcript
siRNA: small interfering RNA
TLR: Toll-like receptor
TNF*α*: tumor necrosis factor *α*
TSA: trichostatin A
VAChT: vesicular acetylcholine transporter
